# Workable male sterility systems for hybrid rice: Genetics, biochemistry, molecular biology, and utilization

**DOI:** 10.1186/s12284-014-0013-6

**Published:** 2014-08-13

**Authors:** Jian-Zhong Huang, Zhi-Guo E, Hua-Li Zhang, Qing-Yao Shu

**Affiliations:** State Key Laboratory of Rice Biology, Institute of Nuclear Agricultural Sciences, Zhejiang University, Hangzhou, 310029 China; China National Rice Research Institute, 28 Shuidaosuo Road, Fuyang, 311401 Zhejiang, China

**Keywords:** Cytoplasmic male sterility (CMS), Environment-conditioned genic male sterility (EGMS), Restorer of fertility (Rf), Chimeric mitochondrial gene, Cytotoxic protein, Pentatricopeptide repeat (PPR)

## Abstract

The exploitation of male sterility systems has enabled the commercialization of heterosis in rice, with greatly increased yield and total production of this major staple food crop. Hybrid rice, which was adopted in the 1970s, now covers nearly 13.6 million hectares each year in China alone. Various types of cytoplasmic male sterility (CMS) and environment-conditioned genic male sterility (EGMS) systems have been applied in hybrid rice production. In this paper, recent advances in genetics, biochemistry, and molecular biology are reviewed with an emphasis on major male sterility systems in rice: five CMS systems, i.e., BT-, HL-, WA-, LD- and CW- CMS, and two EGMS systems, i.e., photoperiod- and temperature-sensitive genic male sterility (P/TGMS). The interaction of chimeric mitochondrial genes with nuclear genes causes CMS, which may be restored by *restorer of fertility* (*Rf*) genes. The PGMS, on the other hand, is conditioned by a non-coding RNA gene. A survey of the various CMS and EGMS lines used in hybrid rice production over the past three decades shows that the two-line system utilizing EGMS lines is playing a steadily larger role and TGMS lines predominate the current two-line system for hybrid rice production. The findings and experience gained during development and application of, and research on male sterility in rice not only advanced our understanding but also shed light on applications to other crops.

**Electronic supplementary material:**

The online version of this article (doi:10.1186/s12284-014-0013-6) contains supplementary material, which is available to authorized users.

## Review

Male reproductive development in plants involves several major developmental stages in series and along several cell lineage pathways, which include specification of stamen primordia, production of sporogenous cells, development of tapetum and microspore mother cells (MMCs), meiosis, formation of free haploid microspores, degeneration of tapetum and release of mature pollen grains (Goldberg et al. [1993]). Arrest of any of these steps can result in male sterility (MS), the failure to produce or release functional pollen grains. The phenotypic manifestations of MS may range from the complete absence of male organs, abnormal sporogenous tissues, to the inability of anther to dehisce or of pollen to germinate on compatible stigma (Chase et al. [2010]).

Evolutionarily, MS has been a subtle means by which plants prevent self-pollination and increase genetic diversity (Hanson [1991]). Over the past century, MS has facilitated the use of heterosis (or hybrid vigor) in crop production. Utilization of heterosis, the superior performance that the first generation (F_1_) hybrid demonstrates over its two parental lines, depends on the cost-effective production of hybrid seeds. Rice is a staple food crop for more than half of the world’s population; the use of heterosis in rice is second only to that in corn, among crop plants, and has played a significant role in further increasing rice yield after the first Green Revolution (Li et al. [2007]).

The success of hybrid rice has greatly promoted the search for and study of MS in rice. Several articles have recently reviewed the key genes and networks that determine male reproductive development, including the differentiation of sporophytic cells (Xing et al. [2011]; Feng et al. [2013]), specification of tapetum and microsporocyte cells (Zhang and Yang [2014]), and biosynthesis and regulation of sporopollenin and pollen exine development (Ariizumi and Toriyama [2011]; Liu and Fan [2013]). Mutations in such genes often result in MS in different forms, e.g. knockout mutation of *CAP1*, which encodes l-arabinokinase, resulted in collapsed abnormal pollens (Ueda et al. [2013]), and microsporeless anthers resulted from null mutations of *MSCA1* in corn (Chaubal et al. [2003]) and *MIL1* in rice (Hong et al. [2012]). As reviewed recently by Guo and Liu ([2012]) and Wang et al. ([2013b]), more than 40 MS genes have been cloned in rice. Shortly after the publication of these two reviews, several more rice fertility/sterility-related genes were reported, including genes underpinning tapetum function and hence pollen development (Liu and Fan [2013]; Ji et al. [2013]), genes required for the development of the anther and pollen (Moon et al. [2013]; Niu et al. [2013a], [b]), and genes for pollen germination and pollen tube growth (Huang et al. [2013b]). Clearly, the list is expected to grow in the near future. Although identifying genes and pathways is necessary in order to understand the underlying mechanisms in the development of the male reproductive system, not all MS mutations have practical use in hybrid crop production. This paper aims to analyze different MS systems that have been explored in hybrid rice production and summarize the latest understanding of their genetics, biochemistry, and biology. We also describe the dynamics of different MS systems in hybrid rice production in China over the past 30 years.

## MS systems used in hybrid rice production

Commercialization of any hybrid crop can only be achieved if reasonably priced technical solutions to hybrid seed production are available. In rice, hybrid seed production was first attempted using chemical hybridizing agent in the 1970s, but this approach was no longer used after MS systems became available. In order for an MS system to be workable for hybrid seed production, it must meet the following prerequisites: (1) complete and stable MS during hybrid seed production; (2) no substantial negative effect on MS and hybrid plants; (3) ability to multiply MS seeds through an intermediate genetic line (maintainer) or under particular environmental conditions; (4) ability to fully achieve fertility in hybrids. Therefore, although a number of MS systems have been generated during the past 40 years, only those that met these requirements were adopted in hybrid production. So far, two distinct systems have been utilized in hybrid rice production: cytoplasmic male sterility (CMS) and environment-conditioned genic male sterility (EGMS).

### CMS systems

Numerous CMS systems with different cytoplasm/nucleus combinations have been generated through backcross breeding. The cytoplasm and nucleus of CMS lines may originate from two different species, two different subspecies (*indica* × *japonica*), or two cultivars (*indica* × *indica*) (Virmani [1994]; Cheng et al. [2007]; Fujii et al. [2010]; Huang et al. [2013a],[b]). According to the China Rice Data Center (http://www.ricedata.cn/variety/), a total of 13 types of CMS lines have been used in developing hybrid cultivars, constituting an annual growing area of more than ~6800 ha in at least 1 year from 1983 to 2012 (data before 1983 are unavailable). The cytoplasm and nucleus sources of these 13 different CMS types are summarized in Table 1, with BT-CMS and Dian1-CMS used in *japonica* and other systems used in *indica* hybrid rice production.

**Table 1:** **Major male sterility systems utilized in hybrid rice production in China** ^**1**^

MS type	Progenitor MS line^2^	Leading MS lines^3^
1 Cytoplasmic male sterility (CMS)		
1.1 BT and BT-like CMS		
1.1.1 BT-CMS	Chinsurah Boro II (*indica*) cytoplasm with Liming (*japonica*) nucleus	Liming A; Xu 9201A
1.1.2 Dian1-CMS	Yunnan high altitude landrace rice (*indica*) cytoplasm with Hongmaoying (*japonica*) nucleus	Yongjing 2A; Ning 67A
1.2 HL-CMS	Red-awned wild rice (*Oryza rufipogon*) cytoplasm with Liantangzao (*indica*) nucleus	Yuetai A; Luohong 3A^4^
1.3 WA-and WA-like CMS		
1.3.1 WA-CMS	Wild abortive rice (*Oryza rufipogon*) cytoplasm with Erjiunan 1 (*indica*) nucleus	Zhenshan 97 A, V 20A
1.3.2 D-CMS	Dissi (*indica*) cytoplasm with Zhenshan 97 (*indica*) Nucleus	D-Shan A, D62A
1.3.3 DA-CMS	Dwarf abortive rice (*Oryza rufipogon*) cytoplasm with Xieqingzao (*indica*) nucleus	Xieqingzao A
1.3.4 GA-CMS	Gambiaca (*indica*) cytoplasm with Chaoyang 1 (*indica*) nucleus	Gang 46A
1.3.5 ID-CMS	Indonesia paddy rice (*indica*) cytoplasm with Zhending 28 (*indica*) nucleus	II 32A, You 1A
1.3.6 K-CMS	K52(*japonica*) cytoplasm with Fenglongzao/Qing’er’ai (*indica*) nulceus	K-17A
1.3.7 LX-CMS	Luihui rice (*indica*) cytoplasm with Zhenshan 97B (*indica*)	Yue 4A
1.3.8 Maxie-CMS	MS mutant of Maweizhan (*indica*) with Xieqingzao (*indica*)	Maxie A
1.3.9 NX-CMS	Selected from F_2_ male sterile plants in the progeny of Wanhui 88 (*indica*) x Neihui 92–4 (*indica*) nucleus	Neixiang 2A, Neixiang 5A
1.3.10 Y-CMS	Yegong (*indica* landrace) cytoplasm with BII44-5 (*indica*) nucleus	Y Huanong A
2 Environment-conditioned genic male sterility (EGMS)		
2.1 PGMS	Nongken 58S, a photoperiod-sensitive genic male sterile (PGMS) mutant of a *japonica* cultivar Nongken 58	7001S, N5088S
2.2 P/TGMS	Photoperiod and temperature sensitive genic male sterile (P/TGMS) derived from Nongken 58S	Pei’ai 64S
2.3 TGMS	Spontaneous temperature sensitive genic male sterile (TGMS) mutants Annong S-1 and Zhu 1S	Guangzhan 63S^5^, XinanS

^1^The pedigree information was acquired from the China Rice Data Center (http://www.ricedata.cn/index.htm) and cross checked with references cited therein.

^2^For CMS lines, the progenitor CMS was always developed by successive backcrossing of the nucleus donor to the cytoplasm donor, e.g., BT-CMS line was developed by backcrossing the cultivar Liming as recurrent parent to Chinsurah Boro II. For EGMS lines, the very progenitor mutant is provided.

^3^Leading lines are the top two MS lines whose hybrids have the largest accumulative planting areas according to China Rice Data Center (http://www.ricedata.cn/index.htm).

^4^Honglian A was the first leading HL-CMS line, from which subsequently derived a series of HL-CMS lines such as Huaai 15A, Congguang 41A, Yuetai A, Lu1A ~ Lu3A, Luohong 3A, Luohong 4A, etc. (Zhu [2000]).

^4^Guangzhang 63S is a typical TGMS line although it was selected from progenies derived from Nongken 58S (Xu et al. [2011]).

Both BT-CMS and Dian1-CMS contain *indica* cytoplasm and a *japonica* nucleus, whereas *indica* hybrid rice cultivars contain cytoplasm of diverse origins, including *O. rufipogon* (e.g., WA-CMS), various *indica* cultivars (e.g., GA-CMS, ID-CMS), and one *japonica* genotype (i.e., K-CMS) (Table 1). It is not difficult to develop *japonica* CMS lines using cytoplasm from *O. rufipogon* or other *indica* lines, but such CMS has no practical use because no restorer lines have been identified in *japonica* rice.

**Figure 1:**
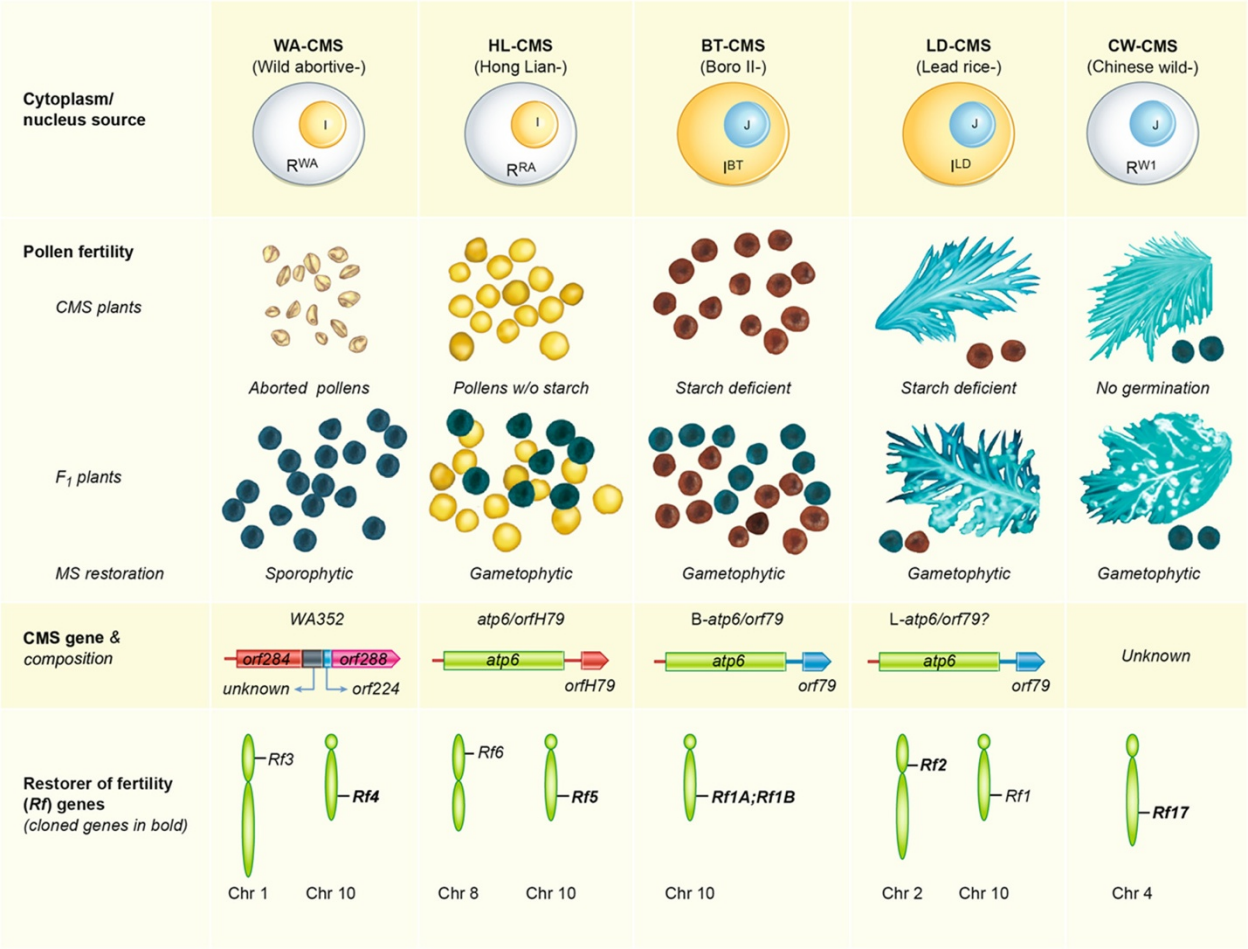
**A schematic presentation of the five well-studied rice CMS types.** Abbreviations for cytoplasm sources are R^WA^ for wild-abortive *Oryza rufipogon*, R^RA^ for red-awned *O. rufipogon*, and R^W1^ for Chinese wild rice (*O. rufipogon*) accession W1; I^BT^ and I^LD^ for *indica* Boro-II type and Lead rice, respectively. Nucleus sources are either *indica* (I) or *japonica* (J).

WA-CMS lines are the most widely deployed lines in hybrid rice production (see below). Pollen abortion in WA-CMS occurs relatively early during microspore development, mainly at the uninucleate stage (Luo et al. [2013]), resulting in amorphous aborted pollen grains (Figure 1). The pollen abortion is determined by the genotype of sporophytic tissues, not by the genotype of the pollen itself. That is, aborted pollens are only produced in plants with homozygous *rf* (restorer of fertility) gene (s) and CMS factor (s), but not in plants that are heterozygous at the *Rf* locus (Figure 1, pollen fertility of F_1_ plants). All other CMS types of *indica* rice, except for HL-CMS, are similar to WA-CMS and are classified as WA-CMS-like types (Table 1).

Pollen development in HL-CMS lines is arrested at the binucleate stage while that of BT-CMS arrested at the trinucleate stage. In contrast to the irregular morphology in WA-CMS, the pollen grains in both HL- and BT-CMS are spherical, and are unstainable or stainable, respectively, in I_2_-KI solution (Li et al. [2007]). Due to their deficiency in starch accumulation, pollen grains of BT-CMS is stained lighter than normal pollen grains (Figure 1; Wang et al. [2006]); the intensity of staining, however, can be rather dark in some BT-CMS lines, almost indiscriminate from that of fertile pollen grains (Li et al. [2007]). Furthermore, unlike in WA-CMS, the MS of the BT- and HL-CMS lines is genetically controlled by gametophytic tissue (i.e., the haploid microspores; hence, only half of the pollen grains in F_1_ plants are viable) (Figure 1). Dian1-CMS lines are very similar to BT-CMS in terms of pollen abortion and fertility restoration; they are classified as BT-like CMS (Table 1).

### EGMS systems

The other MS system that is widely used in hybrid rice breeding is the EGMS system, which includes the photoperiod-sensitive genic male sterility (PGMS) and temperature-sensitive genic male sterility (TGMS) lines. PGMS lines are male-sterile under natural long day conditions and male fertile under natural short day conditions (Ding et al. [2012a]), whereas TGMS lines are sterile at high temperatures and fertile at lower temperatures (Xu et al. [2011]). Some lines, such as Pei’ai 64S, are male sterile under both long day and high temperature conditions and are referred to as P/TGMS lines (Zhou et al. [2012]).

The majority (>95%) of the EGMS lines utilized in hybrid rice production in China were derived from three independent progenitor lines, i.e., PGMS line Nongken 58S (NK58S) and TGMS lines Annong S-1 and Zhu 1S (Si et al. [2012]; Table 1). Many lines derived from NK58S were P/TGMS or even TGMS (e.g., Guangzhan 63S), but the underlying mechanism leading to such dramatic changes has yet to be revealed (Lu [2003]).

### Potential application of other MS systems

Two other CMS types have the potential to be utilized in hybrid rice production. LD-CMS was obtained by Watanabe et al. ([1968]) by performing a backcross of the *japonica* variety Fujisaka 5 to the Burmese rice cultivar ‘Lead Rice’, giving it *indica* cytoplasm and a *japonica* nucleus (Figure 1). The pollen grains of LD-CMS can be slightly stained with I_2_-KI, but they cannot germinate on stigmas (Figure 1). The other CMS type is CW-CMS, which has the cytoplasm of *O. rufipogon* and a *japonica* nucleus. It produces morphologically normal pollen grains that can be stained darkly with I_2_-KI but lacks the ability to germinate (Figure 1; Fujii and Toriyama [2005]). Both LD-CMS and CW-CMS are gametophytically controlled and hence half of the pollen grains of F_1_ plants are viable (Figure 1).

A novel type of EGMS rice, known as rPGMS (reverse PGMS), may also be useful in hybrid rice system. This rice shows normal male fertility under long day conditions (>13.5 h) but is male sterile under short day conditions (<12.5 h). It can be used in a two-line hybrid system by producing hybrid seeds in the tropics and subtropics (e.g., Sanya, Hainan) and multiplying seeds of rPGMS lines under long day conditions (e.g., summer season in Shanghai) (Zhang et al. [2013]).

## Genetics of CMS and fertility restoration

The CMS is controlled by the interaction of cytoplasmic factors (now widely identified as mitochondrial genetic factors) and nuclear genes (Chen and Liu [2014]). As shown in Figure 1, most CMS genes and their corresponding *Rf* genes have already been identified.

### CMS genes

The genetic factors conditioning BT-, HL-, and WA-CMS are all chimeric genes, probably as a result of the rearrangement of the mitochondrial genome (Figure 1). The BT-CMS gene, a mitochondrial open reading frame, *orf79*, was the first CMS gene identified (Akagi et al. [1994]) and subsequently cloned (Wang et al. [2006]) in rice. It is co-transcribed with a duplicated *atp6* and hence is also known as B-*atp6-orf79* (Figure 1). Mitochondrial DNA analysis suggested that *orf79* may also be responsible for Dian1-CMS (Luan et al. [2013]).

In HL-CMS lines, a chimeric ORF defined as *atp6-orfH79* is the gene conditioning MS (Figure 1). Although nucleotide sequences of *orfH79* and *orf79* share 98% identity, the intergenic regions between *atp6-orfH79* and B-*atp6-orf79* are significantly different, suggesting that a*tp6-orfH79* and B-*atp6-orf79* diverged from a common ancestor (Yi et al. [2002]; Peng et al. [2010]; Hu et al. [2012]).

Two differentially expressed transcripts, one of them containing the ribosomal protein gene *rpl5*, were identified by examining the transcripts of the whole mitochondrial genomes of a WA-CMS line, Zhenshan 97A and of its maintainer, Zhenshan 97B (Liu et al. [2007]). The same group recently used *rpl5* to probe the rearranged region in the mitochondrial genome and identified the WA-CMS gene, named *WA352* (*Wild Abortive 352*), which is comprised of three rice mitochondrial genomic segments (*orf284*, *orf224*, and *orf288*) and one segment of unknown origin (Figure 1), and encodes a 352-residue putative protein with three transmembrane segments (Luo et al. [2013]).

Previous work by Bentolila and Stefanov ([2012]), constituting the complete sequencing of male-fertile and male-sterile mitochondrial genomes, identified a WA-CMS-specific ORF, *orf126*, as a plausible candidate for the WA-CMS causative gene. This result is consistent with that of Luo et al. ([2013]) because *orf126* is indeed part of *WA352*. Independently, Das et al. ([2010]) also identified rearrangements around the regions of *atp6* and *orfB*. According to Luo et al. ([2013]), the *atp6* locus is rearranged and directly linked to *WA352*, which is less than 20 kb away from *orfB* in WA-CMS. Therefore, the results of these studies all corroborate one another.

The CMS gene that conditions LD-CMS has yet to be determined, but a B-*atp6-orf79*-like structure (L*-atp6-orf79*) was identified as the candidate (Figure 1). In the mitochondrion of LD-CMS, there is only one copy of *atp6* linked with *orf79*, which is different from BT-CMS and HL-CMS, the mitochondria of which retain a normal *atp6* (N-*atp6*) in its origin position (Itabashi et al. [2009]).

No B-*atp6-orf79*-like structure was identified in the mitochondrion of CW-CMS, and the cytoplasmic factor (s) conditioning pollen sterility has yet to be determined (Fujii et al. [2010]).

### *Restorer of fertility* genes

It has been well documented that CMS can be restored by one or two *Rf* genes. A total of six *Rf* genes (*Rf1a*, *Rf1b*, *Rf2*, *Rf4*, *Rf5* and *Rf17*) have been cloned (Figure 1), and all except *Rf17* are dominant.

Two fertility restoration genes, *Rf1a* and *Rf1b*, both encoding proteins containing pentatricopeptide repeat (PPR) motifs, were identified as being able to restore the fertility of BT-CMS (Kazama and Toriyama [2003]; Akagi et al. [2004]; Komori et al. [2004]; Wang et al. [2006]). Both *Rf1a* and *Rf1b* are located in the classical *Rf1* locus. The *rf1a* allele differs from *Rf1a* due to a frameshift mutation that results in a truncated putative protein of 266 amino acids (Komori et al. [2004]; Wang et al. [2006]). A single-nucleotide polymorphism (SNP) of A^1235^-to-G causes the missense mutation of *Rf1b* to *rf1b* by substituting Asn^412^ for Ser (Wang et al. [2006]).

MS of HL-CMS can be restored by either *Rf5* or *Rf6*, producing 50% normal pollen grains in F_1_ plants (Figure 1). When both *Rf5* and *Rf6* are present, F_1_ plants may have 75% normal pollen grains (Huang et al. [2012]). Recently, the *Rf* 5 gene was cloned and was identified to be the same gene as *Rf1a* or *Rf1*, which encodes the PPR protein PPR791 (Hu et al. [2012]). Sequencing of *Rf5* and *rf5* identified a single nucleotide T^791^-to-A alteration at the fourth PPR motif, which results in a nonsense mutation (TAT to TAA) in the HL-CMS line (Hu et al. [2012]).

WA-CMS can be restored by either *Rf3* or *Rf4*, located on chromosome 1 and 10, respectively (Figure 1). Numerous attempts have been made to delimit and ultimately clone the two genes without much success (Ahmadikhah and Karlov [2006]; Ngangkham et al. [2010]; Suresh et al. [2012]). The breakthrough was not made until very recently by Tang et al. ([2014]), who finally cloned the *Rf4* gene, which also encodes a PPR protein.

Pollen fertility of LD-CMS can be restored by either *Rf1* or *Rf2*; the latter has already been cloned (Figure 1; Itabashi et al. [2009], [2011]). The *Rf2* gene encodes a mitochondrial glycine-rich protein; replacement of isoleucine by threonine at amino acid 78 of the RF2 protein causes functional loss of the *rf2* allele (Itabashi et al. [2011]). The CW-CMS is restored by a single nuclear gene, *Rf17*, which is a *retrograde-regulated male sterility* (*rms*) gene (Figure 1; Fujii and Toriyama [2009]). Contrary to this finding, the same group suggested in earlier reports that two other genes, *DCW11* and *OsNek3*, were related to pollen sterility in CW-CMS rice (Fujii and Toriyama [2008]; Fujii et al. [2009]). It is now evident that diversified mechanisms have been evolved for restoring fertility in CMS with multilayer interactions between the mitochondrial and nucleus genes (Chen and Liu [2014]).

### Relationships between different CMS-Rf systems

In addition to the three major CMS types (i.e., WA-, BT-, and HL-CMS), several other CMS types were bred independently and have different cytoplasm and nucleus sources (Table 1). Further studies revealed that both cytoplasm and nuclear genetic determinants are almost identical among some of them; hence, they may be classified into a common group.

First, the fertility restoration of Dian1-CMS is identical to that of BT-CMS, i.e., restorer lines of the latter are equally effective for the former, although *Rf-D1 (t)* was assigned for Dian1-CMS (Tan et al. [2004]). Subsequent cloning and characterization suggested that *Rf-D1* is highly similar to *Rf1a* and has only one nucleotide difference (Zhu et al. [2009]).

Second, nine CMS types are classified as WA-like CMS (Table 1) on the basis of the following observations: (1) *WA352* is also identified in the GA-, D-, DA-, ID-, K-, and Y-CMS lines (Luo et al. [2013]); (2) *Rf3* and *Rf4* are effective for restoring the fertility of D-, DA-, ID-, GA-, Y-, and WA-CMS (Sattari et al. [2008]; Cai et al. [2014]); (3) these nine CMS types possess common mitotype-specific sequences that differ from fertile genotypes and from other CMS systems (e.g., BT-CMS, HL-CMS) (Xie et al. [2014]); and (4) they have identical or highly similar mitochondrial DNA (Luan et al. [2013]). However, we should not exclude the possibility that differences exist in their mitochondrial genomes. For example, Xu et al. ([2013]) recently indicated that male sterile cytoplasm has a marked effect on DNA methylation, which is enhanced to a much greater extent in WA- and ID-CMS than in G- and D-CMS.

Third, restorer lines containing *Rf4* can often restore the fertility of BT-CMS and HL-CMS (but the opposite is not true). This effect might be explained by the following considerations: (1) Plants with *Rf4* may also possess *Rf1a* and *Rf1b*. (2) The *Rf4* allele has more functions than *Rf1,* and *Rf4* itself has the ability to restore the fertility of both WA-CMS and BT-CMS. Notably, the recent cloning of *Rf4* reveals that it also encodes a PPR protein, with high amino acid sequence identity with Rf1a of BT-CMS (Tang et al. [2014]).

## Genetic control of EGMS

### First and second photoperiod/temperature reaction systems in rice

Rice is a short-day plant; short day length accelerates panicle initiation and promotes flowering, but long day length delays or inhibits development. Likewise, relatively high temperatures promote rice growth and development. This reaction of plants to photoperiod and temperature is described as the first photoperiod/temperature reaction (FPTR, Yuan et al. [1993]). The P/TGMS lines described in this paper are those in which the male reproductive system responds to both day length and temperature, in the so-called second photoperiod/temperature reaction (SPTR).

Different EGMS lines may have very different fertility responses to photoperiod and temperature. Cheng et al. ([1996]) classified EGMS lines into three types: PGMS lines respond to either photoperiod or photoperiod-and-temperature, but not to temperature alone; TGMS lines respond to temperature, but not to photoperiod; P/TGMS lines are characterized by responding to photoperiod-and-temperature for their fertility transition.

### Genes underpinning EGMS in rice

During the past 20 years, a number of EGMS lines have been identified that show genic MS under different conditions: long day (PGMS) or short day (reverse PGMS, rPGMS), high temperature (TGMS) or low temperature (rTGMS), and either long day or high temperature. In all these cases, the pollen fertility of EGMS systems is sporophytically controlled by nuclear gene (s), and the loci that control PGMS or TGMS, including rPGMS or rTGMS, have been mapped to different chromosomes (Si et al. [2012]; Sheng et al. [2013]; Zhang et al. [2013]). These mappings include PGMS genes: *pms1*, *pms2*, *pms3*; rPGMS genes: *rpms1*, *rpms2*, *csa*; TGMS genes: *tms1*, *tms2*, *tms3*, *tms4*, *tms5*, *tms6*, *tms6(t)*, *tms9*; and P/TGMS genes: *p/tms12-1*, *pms1(t)*. Some of these genes may be allelic and two of them, *pms3* (*p/tms12-1*) (Ding et al. [2012a]; Zhou et al. [2012]) and *csa* (Zhang et al. [2013]), have been cloned.

#### PGMS and P/TGMS

NK58S, the first PGMS, was identified in 1973 from a Nongken58 population. It exhibits complete MS when growing under long days (day length more than 13 h), but complete or partial fertility under short days (day length less than 13 h) (Zhang and Yuan [1989]). However, Pei’ai 64S, developed from a cross between NK58S and Pei’ai 64 followed by backcrossing with Pei’ai 64, showed MS under both long day and high temperature conditions (Luo et al. [1992]). W6154S, also derived from NK58S, is a TGMS line. Zhang et al. ([1994]) identified two genes underlying the PGMS of NK58S. A study on the allelism of gene (s) for P/TGMS lines further showed that there were allelic male sterile genes between NK58S and its derivatives W6154S and Pei’ai 64S, but male sterile genes from the latter two are nonallelic, suggesting that NK58S has at least two genes underpinning its PGMS (Li et al. [2003]). Two recent independent studies identified the identical causative SNP for both the PGMS of NK58S (*pms3*, Ding et al. [2012a]) and the TGMS of Pei’ai 64S (*p/tms12-1*, Zhou et al. [2012]), although the identity of the locus containing the SNP was different (see below).

An rPGMS gene, *carbon starved anther* (*csa*), was recently cloned and may be potentially useful for diversification of the two-line hybrid rice system (Zhang et al. [2013]).

#### TGMS

Several spontaneous TGMS mutants have been independently identified in breeding programs; more TGMS lines were selected in the progenies derived from NK58S (Si et al. [2012]). Genetic analyses indicated that the TGMS trait is under the control of single recessive genes. Among the fine-mapped TGMS genes, those of Annong S-1 (*tms5*), Guangzhan 63S (*ptgms2-1*), and Zhu 1S (*tms9*) are all located on chromosome 2. Whereas *tms5* and *ptgms2-1* were delimited to a partially overlapped region, *tms9* was fine-mapped to a different segment near that of *ptgms2-1*/*tms5* (Sheng et al. [2013]). Candidate genes were proposed for *tms5* (*OsNAC6*; Yang et al. [2007]) and *ptgms2-1* (a ribonuclease Z homolog, *RNZ*; Xu et al. [2011]), but none were suggested for *tms9* (Sheng et al. [2013]). Our recent study, however, demonstrated that Annong S-1, Guangzhan 63S and Zhu 1S carry allelic TGMS genes (i.e. *tms5*, *ptgms2-1*, and *tms9* are allelic), and further characterization of more than 300 non-EGMS and EGMS lines suggested that an identical nonsense mutation of the *RNZ* gene, i.e. *RNZ*^*m*^.conditions the TGMS of Guangzhan 63S, Zhu 1S, Annong S-1, and a number of other TGMS lines (Zhang et al. [2014]).

## Cytology, biochemistry, and molecular biology of MS

### Male reproductive development and tapetal PCD

Anther development in rice occurs over 14 stages (Zhang and Wilson [2009]), and the specification, development, and degradation of the anther are tightly regulated by various genes and pathways. Dysfunction of any gene may result in MS (Suzuki [2009]; Wilson and Zhang [2009]; Ariizumi and Toriyama [2011]; Feng et al. [2013]).

**Figure 2:**
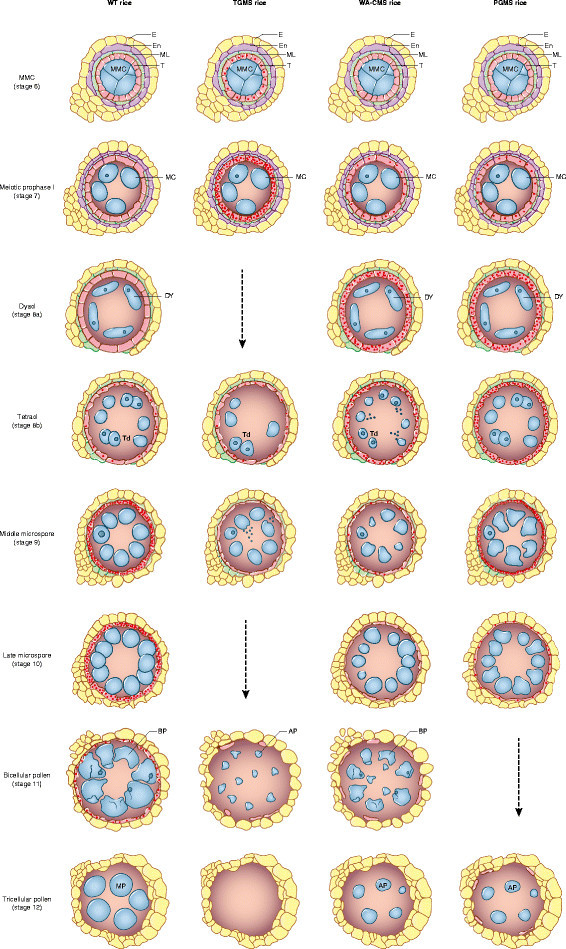
**A schematic presentation of anther and pollen development in wild type (WT) rice, wild-abortive CMS (WA-CMS) rice, temperature- and photoperiod -sensitive genic male sterile (TGMS and PGMS) rice.** Stage demarcation and developmental features of WT rice are adopted from Zhang and Wilson ([2009]); those of WA-CMS, TGMS and PGMS are according to Luo et al. ([2013]), Ku et al. ([2003]), and Ding et al. ([2012a]), respectively. Dots represent the DNA fragmentations detected by TUNNEL assay in tapetal cells undergoing programmed cell death. AP, aborted pollen; BP, binucleate pollen; E, epidermis; En, endothecium; ML, middle layer; T, tapetum; MMC, microspore mother cell; MC, meiotic cell; DY, dyad; Td: tetrad; MP, mature pollen.

The development of pollen and degradation of the endothecium, middle layer, and tapetal cells are illustrated in Figure 2. The tapetum is the nursing tissue inside the anther and plays a crucial role in the formation and development of pollen grains (Suzuki [2009]; Ariizumi and Toriyama [2011]). In wild-type plants, tapetum undergoes cellular degeneration by programmed cell death (PCD) and completely disappears by the time the mature pollen grains form. PCD is often observed in anther tissues by terminal deoxynucleotidyl transferase-mediated dUTP nick-end labeling (TUNEL) assay. Slight differences have been reported regarding the commencement of tapetal PCD in rice: One group (Ji et al. [2013]; Luo et al. [2013]) detected PCD as early as stage 8a (the dyad stage), whereas others (Li et al. [2006]; Ding et al. [2012a]) observed the earliest PCD occurring at stage 8b (the tetrad stage) or noted that it peaked at stage 9 (young microspore stage). The correct timing of tapetal PCD is important, and premature or delayed PCD is often associated with MS. Unlike most other rice MS mutants, which have delayed tapetal PCD (Li et al. [2006]; Ji et al. [2013]), certain EGMS and WA-CMS rice have premature tapetal PCD (Ding et al. [2012a]; Luo et al. [2013]; Figure 2).

The TGMS lines of Annong S-1, Xian 1S, and Guangzhan 63S have empty anthers (Ku et al. [2003]; Peng et al. [2010]; Xu et al. [2011]). Premature tapetal PCD initiates as early as the microspore mother cell (MMC) stage (stage 6) and continues until the tapetal cells are completely degraded in Annong S-1 grown under high temperature conditions (Ku et al. [2003]). The premature tapetal PCD resulted in early degradation of the tapetum, causing a decline in the supply of nutrition and other components (e.g. sporopollenin) to microspores, which were ruptured around stage 9. Consequently, no pollen grains were seen in the pollen sac in TGMS lines (Figure 2).

Analysis of the PGMS line NK58S grown under long-day conditions demonstrated that tapetal PCD was already apparent at stage 7 and became intense from stage 8a to stage 9, much earlier than in NK58 (Ding et al. [2012a]). The premature tapetal PCD in NK58S resulted not only in pollen abortion but also incomplete degradation of tapetal cells at later stages (Figure 2).

The different timings of premature tapetal PCD in TGMS and PGMS lines entail distinct consequences on pollen development in these two types (i.e., no pollen is formed in the pollen sac in TGMS lines and pollen abortion occurs in PGMS lines) (Figure 2). However, it remains unclear whether the premature tapetal PCD is induced under MS-inducing conditions, because neither the PGMS gene nor the TGMS gene is involved directly or indirectly in any known PCD pathway.

In WA-CMS line Zhenshan 97A, tapetal PCD was also observed as early as stage 7 (Figure 2), although it was not detected until stage 8a in its maintainer line Zhenshan 97B (Luo et al. [2013]). Tapetal PCD in WA-CMS rice started at the same stage as in PGMS rice, however, TUNEL assay indicated that DNA fragmentation only persisted to stage 9 in tapetal cells. Degradation of tapetal cells started as early as stage 8b, at which stage cytological observation showed debris was leaking from tetrads or tapetal cells. Consequently, tapetal cells degraded earlier than in wild type rice, and abnormal development of microspores could already be seen at stage 9 (Luo et al. [2013]; Figure 2). The molecular mechanism leading to premature tapetal PCD in WA-CMS rice is well explained (see below).

### Molecular basis of CMS and its restoration

#### BT-CMS: cytotoxicity and detoxification

In the BT-CMS system, CMS is known to be caused by a cytotoxic peptide, ORF79, encoded by a mitochondrial dicistronic gene B-*atp6-orf79*. ORF79 is a transmembrane protein; it is toxic to *Escherichia coli* (Wang et al. [2006]) and is also toxic to plant regeneration when it targets the mitochondria (Kojima et al. [2010]). ORF79 is accumulated specifically in microspores, despite its constitutive expression (Wang et al. [2006]), which provides a tight correlation between its accumulation and the phenotype of gametophytic MS. The molecular mechanism that regulates the expression of ORF79 and the way in which it causes the arrest of microspore development at the trinucleate stage are unknown.

BT-CMS is restored by two related PPR motif genes, *Rf1a* and *Rf1b*, by blocking ORF79 production through distinct modes of mRNA silencing: endonucleolytic cleavage of the dicistronic B-*atp6*-*orf79* mRNA by RF1A and degradation by RF1B. In the presence of these two restorers, the *Rf1a* gene has an epistatic effect over the *Rf1b* gene in mRNA processing (Wang et al. [2006]). Further studies suggested that the RF1 protein mediates cleavage of the dicistronic mRNA by binding to the intergenic region, and the processed *orf79* transcripts are degraded and unable to associate with ribosome. As a result, the *orf79* expression is drastically reduced due to the processing of *atp6-orf79* transcripts (Kazama et al. [2008]).

#### HL-CMS: mitochondrion dysfunction

The mitochondrial dicistronic gene *atp6-orfH79* is responsible for HL-CMS (Peng et al. [2010]), as proposed by Wang et al. ([2002]). Transcripts of *orf79* and *orfH79* differ in only five nucleotides, each of which results in distinctly different codon (Peng et al. [2010]). Like *orf79*, *orfH79* is constitutively expressed; however, accumulation of ORFH79 is not limited to microspores as it is for *orf79* in BT-CMS. Rather, it is accumulated mainly in the mitochondria in both vegetative and reproductive tissues, preferentially in sporogenous cells and root tips (Peng et al. [2010]). ORFH79 impairs mitochondrial function through its interaction with P61, a subunit of electron transport chain (ETC) complex III in HL-CMS rice (Wang et al. [2013a]). The interaction of ORFH79 and P61 significantly reduces the activity of ETC III through an as-yet-unknown mechanism, impairs the electron transport efficiency, and down-regulates the production of ATP. Concomitantly, more reactive oxygen species (ROS) are produced accompanying increased electron leakage from the ETC (Wang et al. [2013a]). The observations of increased ROS and preferential accumulation of ORFH79 in sporogenous cells are in accordance with a study that detected PCD in microspores of the HL-CMS line Yuetai A (Li et al. [2004]).

Unlike the RF1A-binding to B-atp6-orfH79 transcript, RF5 (the same protein of RF1A) is unable to bind to atp6-orfH79 transcript directly, due to its divergent intergenic region. Instead, a RF5’s partner protein, GRP162, can bind to the *atp6-orfH79* through an RNA recognition motif. These two proteins interact physically with each other in the so-called restoration of fertility complex (RFC), which can cleave *atp6-orfH79* at a site 1169 nucleotides away from the *atp6* start codon (Hu et al. [2012]). Additional components are predicted to participate in the RFC, because neither RF5 nor GRP162 can cleave the mRNA; it remains to be determined which factor of the RFC possesses the capacity as an endoribonuclease to process *atp6-orfH79*.

Another gene, *Rf6*, can also restore the fertility of HL-CMS, but little is known regarding its identity or the mechanism leading to fertility restoration (Huang et al. [2012]).

#### WA-CMS: a protein causing premature tapetal PCD

MS in WA-CMS rice is caused by WA352, which interacts with a nuclear-encoded integral protein of the inner mitochondrial membrane, OsCOX11. COX11 proteins are essential for the assembly of cytochrome c oxidase; they display high levels of conservation among eukaryotes and play a role in hydrogen peroxide degradation (Banting and Glerum [2006]). A significantly increased amount of ROS was observed in the tapetum of WA-CMS line Zhenshan 97A, but not in its maintainer, at the MMC stage (Luo et al. [2013]). Hence, it is assumed that the elevation of ROS in WA-CMS line, as a result of the interaction of WA352 with OsCOX11, prevents the normal function of OsCOX11 in H_2_O_2_ degradation. The excessive amount of ROS could further affect the mitochondrial membrane permeability and promote Cyt *c* release into the cytosol, triggering PCD (Luo et al. [2013]).

Both *OsCOX11* and *WA352* are constitutively expressed; however, while OsCOX11 protein is accumulated in all tissues, WA352 protein was detected only in anthers, not in leaves. In the anthers, WA352 was observed mainly in tapetal cells at the MMC stage and diminished after the meiotic prophase I stage. The tissue specificity and accumulation duration of WA352 are in good accordance with the occurrence of tapetal PCD as detected by TUNEL assay, the earliest PCD being observed as early as stage 7 of anther development (Figure 2; Luo et al. [2013]). However, it is not known why WA352 only accumulates in tapetal cells at the MMC stage. Further studies are needed to uncover the molecular mechanism and genetic factor (s) regulating time-specific protein accumulation.

WA-CMS can be restored by either *Rf3* or *Rf4* (Figure 1). The amounts of *WA352* transcripts in the *Rf4*-carrying lines with WA-CMS cytoplasm were decreased to ~20–25% of those in the WA-CMS line without *Rf4*, but were not affected in the *Rf3*-carrying lines. WA352 was undetectable in either *Rf3*- or *Rf4*-carrying young anthers (Luo et al. [2013]). These observations suggest different mechanisms of male fertility restoration be deployed by the two *Rf* genes: RF4 may cleave the *WA352* transcript and RF3 may suppress its translation. In this regard, RF4 may function like that of RF1B, which mediates the degradation of atp6-orf79 mRNA, whereas RF3’s mode of action would be distinctly different from those of RF1A and RF1B (see above).

#### LD-CMS and CW-CMS: CMS genes unknown, restorer genes cloned

Fertility of the LD-CMS can be restored by either *Rf1* or *Rf2* (Figure 2). Although LD-CMS rice also possesses a chimeric *atp6-orf79* dicistronic gene, *L-atp6-orf79* (Figure 2), the CMS in LD-cytoplasm is not caused by the accumulation of ORF79. The induction and restoration of LD-CMS are different from those in BT-CMS (Itabashi et al. [2009]). The *Rf2* gene has already been cloned and is known to encode a mitochondrial glycine-rich protein, but the mechanism of CMS restoration has yet to be determined (Itabashi et al. [2011]).

As in LD-CMS, the cytoplasmic genetic factor that causes MS in CW-CMS has not been identified. However, its restorer of fertility gene, *Rf17*, is known to encode a 178-aa mitochondrial protein of unknown function. *Rf17* is considered to be an *rms* gene, because its expression is regulated by the cytoplasmic genotype. The low expression of RMS in a restorer line of CW-CMS, probably due to a SNP in its promoter region, is speculated to restore compatibility between the nucleus and mitochondria, leading to male fertility (Fujii and Toriyama [2009]).

### Molecular genetics and biology of EGMS

#### P/TGMS results from a noncoding RNA mutation

As mentioned above, a noncoding RNA was recently identified to underpin the PGMS of NK58S (*pms3*) and TGMS of Pei’ai 64S (*p/tms12-1*), with a common C → G SNP as the causative element of P/TGMS (Ding et al. [2012a]; Zhou et al. [2012]). However, the functional element of this locus and its role in P/TGMS development were elucidated quite differently by the two groups.

Ding et al. ([2012a]) showed that the locus encodes a long noncoding RNA (lncRNA) designated LDMAR (long day-specific male fertility associated RNA), and they argued that a sufficient amount of LDMAR is essential for male fertility under long day conditions. The low abundance of LDMAR transcripts, rather than the C → G SNP, is responsible for the PGMS of NK58S, because overexpression of the LDMAR transcript of NK58S restored the fertility of NK58S under long day conditions. They indicated that the low expression of LDMAR in NK58S is due to increased methylation in the promoter region, compared with NK58 (Ding et al. [2012a]). In a later study, they identified in the promoter region of LDMAR a siRNA called Psi-LDMAR, which is more abundant in NK58S than its wild type line (Ding et al. [2012b]). They suggested that the enhanced methylation in the LDMAR promoter region induced by the greatly enriched Psi–LDMAR repressed the expression of LDMAR. However, several puzzles remain: First, as the authors noted, Psi-LDMAR is produced mainly in leaves, but regulation of fertility should reside in panicles (Ding et al. [2012b]); Second, the role of the C → G SNP in increasing methylation of the promoter directly, or indirectly through the generation of Psi-LDMAR, was not addressed.

After identifying the lncRNA locus, Zhou et al. ([2012]) further narrowed down its functional form to a small, 21-nt RNA, designated as osa-smR5864w and osa-smR5864m for the wild-type and mutant allele, respectively. The small RNA may be a product of a 136-nt intermediate precursor. They speculated that osa-smR5864w may be the functional form and regulate male development under sterility-inducing conditions by cross-talking between the genetic networks and environmental conditions. However, no gene known to be involved in anther and pollen development has been shown to be the target of osa-smR5864w.

In addition to offering different explanations for the functional identity of the lncRNA locus, Ding et al. ([2012a]) and Zhou et al. ([2012]) made the following different observations: (1) LDMAR is expressed in all tissues and is relatively higher in panicles, whereas osa-smR5864w is mainly expressed in panicles; (2) Expression of LDMAR in NK58 is significantly higher under long days than under short days, and is significantly higher in NK58 than in NK58S under any day length, while expression of osa-smR5864w is almost independent of growing conditions. Consequently, Ding et al. ([2012a]) argued that occurrence of PGMS under long day resulted from lower expression of LDMAR rather than from the C → G SNP; Zhou et al. ([2012]) inferred that it was the function rather than the amount of osa-smR5864w that determined PGMS in NK58S and TGMS in Pei’ai 64S.

Further studies will verify which hypothesis is correct, but the authors of this review are inclined to agree with Zhou et al. ([2012]) for the following reasons. (1) The functional importance of the C → G SNP is explained in osa-smR5864w and osa-smR5864m, but it is very speculative in LDMAR. (2) The spatial expression of osa-smR5864w is more relevant to its function than is the spatial expression of LDMAR. (3) The possibility that LDMAR is a precursor of small RNA was not excluded. Indeed, Ding et al. ([2012a]) predicted and verified by RT-PCR that three small RNAs could be processed from a stem-loop structure involving 145 bases of LDMAR, and the smRNA-1 with the C → G SNP is exactly the same as osa-smR5864.

#### TGMS may be due to null mutation of *OsaTRZ1*

The RNase Z enzyme is a highly conserved single-chain endoribonuclease that is expressed in all living cells. There are two classes of RNase Z proteins, long RNase Z^L^ and short RNase Z^S^ (Vogel et al. [2005]). RNase Z catalyzes the hydrolysis of a phosphodiester bond, producing 3’-hydroxy and 5’-phospho termini as it participates in tRNA maturation by cleaving off a 3’ trailer sequence (Mayer et al. [2000]). The first RNase Z gene was cloned from *Arabidopsis* (Schiffer et al. [2002]); studies of homologous genes in various species have revealed that RNase Z could cleave a broader spectrum of substrates, including coding and noncoding RNAs (Xie et al. [2013]).

In plants, RNase Z is described using a prefix for the species, followed by TRZ (e.g., *AthTRZ* and *OsaTRZ* are the RNase Z genes in *Arabidopsis* and rice, respectively) (Fan et al. [2011]). The rice genome has three RNase Z genes: *OsaTRZ1* (*LOC_Os02g12290*) and *OsaTRZ2* (*LOC_Os09g30466*) encoding RNase Z^S^, and *OsaTRZ3* (*LOC_Os01g13150*) encoding RNase Z^L^ (Fan et al. [2011]). *OsaTRZ2* contributes to chloroplast biogenenesis and homozygous *OsaTRZ2* mutants are albino with deficient chlorophyll content due to the arrest of chloroplast development at an early stage (Long et al. [2013]). As indicated above, a nonsense mutation of *OsaTRZ1* (*RNZ*^*m*^) could be responsible for the TGMS traits in rice (Zhang et al. [2014]). Although it is unclear how this mutation leads to TGMS, the following observations in other species suggest a logical pathway by which the *RNZ*^*m*^ mutation could result in TGMS. First, the *Arabidopsis* genome has four RNase Z genes—*AthTRZ1* and *AthTRZ2* for RNase Z^S^, and *AthTRZ3* and *AthTRZ4* for RNase Z^L^—but only the chloroplast-localized *AthTRZ2* is essential. Deletions of the other three are not lethal (Canino et al. [2009]), suggesting that the null mutation of *OsaTRZ1* will also not be lethal for rice development, a phenomenon that fits *RNZ*^*m*^ mutants. Second, it has been proven that conditional knockout at gametogenesis of *Drosophila RNZ* leads to thinner testes and lack of post-meiotic germ cells (Xie et al. [2013]), a phenomenon similar to that observed in TGMS rice: premature degeneration of tapetal cells and lack of pollen in the pollen sac (Figure 2).

Because the function of TRZ genes has been assigned recently, very limited references are available for a thorough judgment of the possible functions of *OsaTRZ1* and its involvement in male gametophyte formation. Further studies are needed to unveil the molecular mechanism of TGMS and to elucidate the functions and working mechanisms of TRZ1 genes in plants in general and in rice in particular.

### Epigenetic regulation of MS and restoration

Epigenetic regulation has recently been identified to play an important role in gene expression. DNA methylation is known to play a role in fertility transformation of rice P/TGMS (Ding et al. [2012b]). In addition, Chen et al. ([2014]) further observed that the DNA methylation level of P/TGMS line Pei’ai 64S was lower under low temperatures and short-day conditions (associated with fertility) than under high temperatures and long-day conditions (associated with sterility), suggesting that DNA methylation may be involved in the sterility–fertility transition of Pei’ai 64S in two different environmental profiles. Similarly, Xu et al. ([2013]) detected DNA methylation sites that were specific to CMS lines or maintainer lines (B lines), implying a specific relationship between DNA methylation at these sites and male-sterile cytoplasm, as well as a relationship with MS. Furthermore, Xu et al. ([2013]) demonstrated that DNA methylation was markedly affected by male-sterile cytoplasms (i.e., WA- and ID-type cytoplasms affected methylation to a much greater degree than did G- and D-type cytoplasms, although there were few differences at the DNA level). Therefore, studies on epigenetic regulation may increase our understanding of the mechanisms regulating MS and restoration.

## Utilization of different rice MS systems in China (1983–2012)

Since the first WA-CMS-based hybrid rice was commercialized in the 1970s in China, several hundred CMS and EGMS lines have been developed, and some of them are currently or were once used in rice production. Although it is known that WA-CMS is the most widely used CMS in China (Cheng et al. [2007]) and in India (Khera et al. [2012]), so far no report has documented the dynamic changes of different MS systems in rice production. The China Rice Data Center (http://www.ricedata.cn/) has kept records of the annual planting area of rice cultivars grown in areas of at least ~6800 ha from 1983 to the present day. Therefore, we are able to analyze the growing areas under hybrid rice cultivation over the past 20 years (1983–2012). The following is the information extracted from the original data.

**Figure 3:**
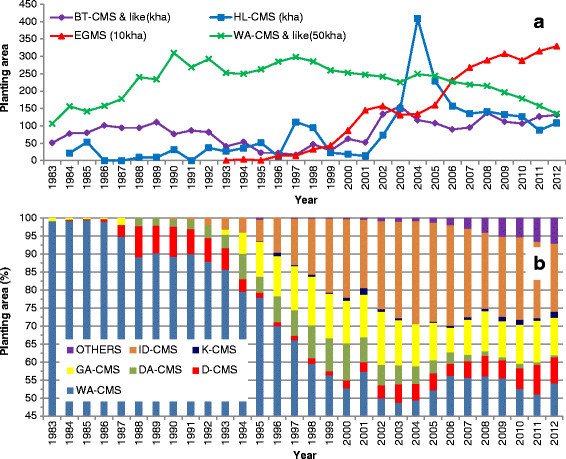
**Planting areas covered by different types of hybrid rice in China (1983–2012).** ***a***, Hybrids based on BT-, HL-, and WA-CMS lines as well as EGMS (environment-conditioned genic male sterility). ***b***, Hybrids based on different CMS types with similar features to WA-CMS. For definition of different CMS types see Table 1. Note the data were composed of hybrid rice cultivars that had grown in more than 50,000 ha (1983 to 2012) in this figure, cultivars with less growing area were not included.

Two-line system hybrid rice was not commercialized until 1993; however, it has since played a steadily larger role in hybrid rice production (Figure 3a). In 2012, two-line system hybrid rice already covered a total growing area of ~3.3 million ha, about one-third of the total hybrid rice growing area (~10 million ha) (Figure 3a) (Note: only the hybrids that had been grown in areas more than 50,000 ha were included in Figure 3).

In order to avoid the genetic vulnerability such as the crop failure of hybrid corn based on T-CMS in the 1970s, Chinese rice breeders from the very beginning have been trying to develop new types of CMS lines and to diversify the cytoplasm sources of these lines. Hence, ~15 new CMS sources other than WA-CMS have been developed and deployed in hybrid rice production. These sources may be classified into three primary groups: BT- and BT-like CMS, HL-CMS, and WA- and WA-like CMS (Table 1).

BT-CMS-based *japonica* hybrid rice was successfully developed in the 1970s, only a few years after WA-CMS-based *indica* hybrids. However, the planting area was very limited compared with the latter (Figure 3a). Within the BT- and BT-like category, Dian1-CMS hybrids are steadily replacing BT-CMS hybrids; the former now comprise ~90% of cultivation (data not shown).

Within the WA-CMS and the WA-CMS-like categories, there are more than a dozen subtypes of CMS lines. Although WA-CMS still dominates among the subtypes, its absolute dominance has been diminishing since the mid-1990s, and now it represent less than 55% of the total CMS-based hybrid rice (Figure 3b). Indeed, this category represents almost the same proportion of all CMS rice because BT- and HL-CMS have a very low percentage of the total CMS (Figure 3a).

## Conclusions

CMS was used initially in the development of hybrid rice in the so-called three-line hybrid system, but EGMS is becoming more popular in hybrid rice production since the two-line hybrid system, in which the EGMS lines are used, has advantages of a wider range of restoring lines, more freely combinations and simple breeding program. CMS is conditioned by chimeric recombinant mitochondrial genes; the fertility of CMS lines may be restored by *Rf* genes. EGMS is underpinned by genes for non-coding RNA, transcriptional factors and RNA-processing enzymes. Different MS systems for rice have undergone dynamic changes in practical application in China.

## Authors’ original submitted files for images

Below are the links to the authors’ original submitted files for images. Authors’ original file for figure 1 Authors’ original file for figure 2 Authors’ original file for figure 3

## Abbreviations

B line: Maintainer line
CMS: Cytoplasmic male sterility
csa: Carbon starved anther
EGMS: Environment-conditioned genic male sterility
ETC: Electron transport chain
F_1_: First generation
FPTR: First photoperiod/temperature reaction
LDMAR: Long day specific male fertility associated RNA
lncRNA: Long non-coding RNA
lncR^m^: lncR with C-to-G SNP that underpins the PGMS phenotype
MMCs: Microspore mother cells
MS: Male sterility
NK58: Nongken58
P/TGMS: Photoperiod-and temperature-sensitive genic male sterility
PCD: Programmed cell death
PGMS: Photoperiod-sensitive genic male sterility
PPR: Pentatricopeptide repeat
Rf: *Restorer of fertility* gene
RFC: Restoration of fertility complex
RMS: Retrograde-regulated male steriity
RNZ: Ribonuclease Z homolog
RNZ^m^: OsaTRZ1 carrying a null mutation that underpins the TGMS phenotype
ROS: Reactive oxygen species
rPGMS: Reverse PGMS
rTGMS: Reverse TGMS
SNP: Single-nucleotide polymorphism
SPTR: Second photoperiod/temperature reaction
TGMS: Temperature-sensitive genic male sterility
TUNEL: Terminal deoxynucleotidyl transferase-mediated dUTP nick-end labeling
