# Supplementary figures and images for: Workable male sterility systems for hybrid rice: Genetics, biochemistry, molecular biology, and utilization
Source: Rice (N Y). 2014 Aug 13;7:13. doi: 10.1186/s12284-014-0013-6 (PMC4883997; doi:10.1186/s12284-014-0013-6)

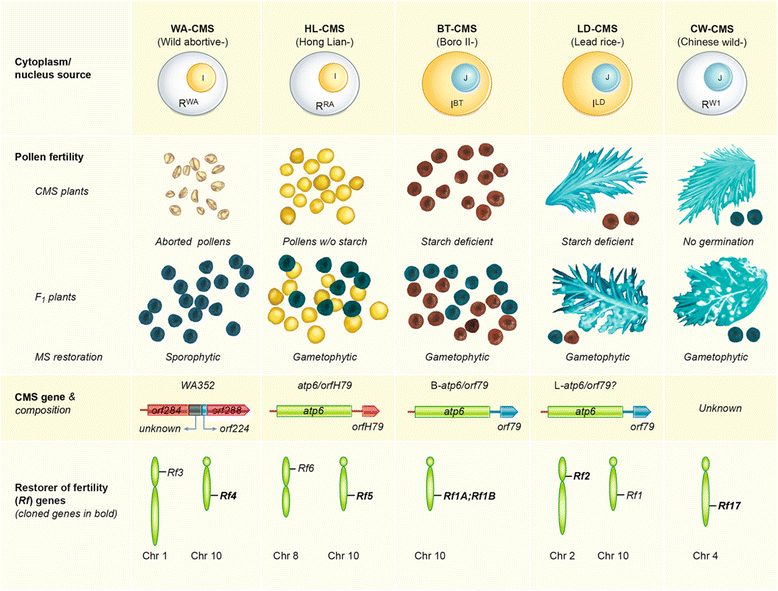

Supplement: Supplementary file 1 — Authors’ original file for figure 1 [file 12284_2014_13_MOESM1_ESM.gif]

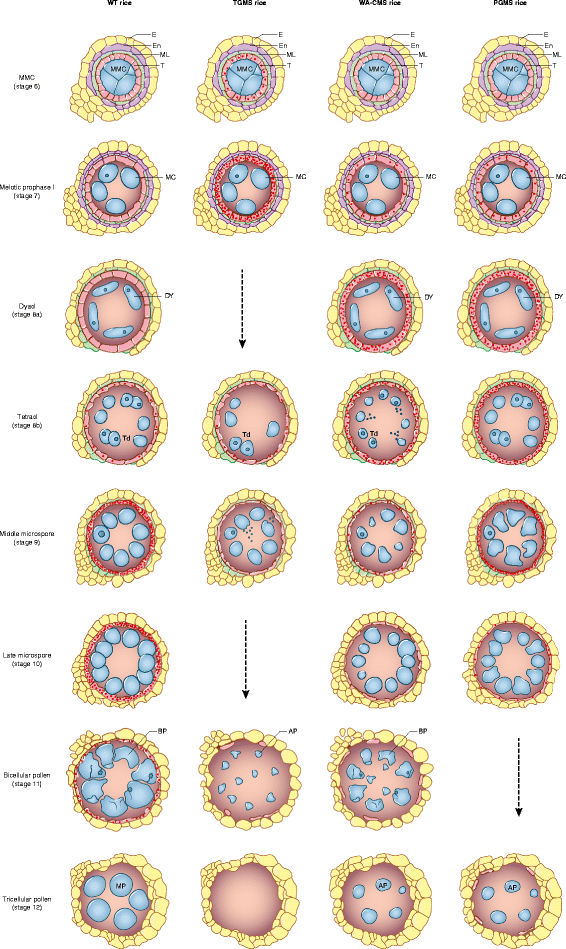

Supplement: Supplementary file 2 — Authors’ original file for figure 2 [file 12284_2014_13_MOESM2_ESM.gif]

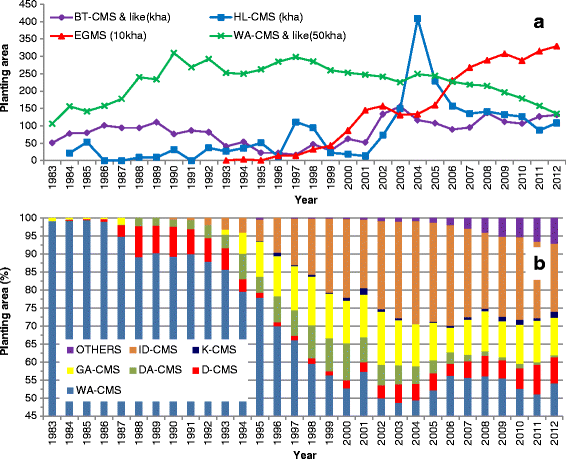

Supplement: Supplementary file 3 — Authors’ original file for figure 3 [file 12284_2014_13_MOESM3_ESM.gif]
